# Urine Biopsy—Liquid Gold for Molecular Detection and Surveillance of Bladder Cancer

**DOI:** 10.3389/fonc.2019.01266

**Published:** 2019-11-19

**Authors:** Uttam Satyal, Abhishek Srivastava, Philip H. Abbosh

**Affiliations:** ^1^Molecular Therapeutics Program, Fox Chase Cancer Center, Philadelphia, PA, United States; ^2^Division of Urologic Oncology, Department of Surgical Oncology, Fox Chase Cancer Center, Philadelphia, PA, United States; ^3^Department of Urology, Albert Einstein Medical Center, Elkins Park, PA, United States

**Keywords:** urine biopsy, bladder cancer, cancer surveillance, prognosis and diagnosis, precision medicine, next generation sequencing

## Abstract

With recent advancements in a non-invasive approach to cancer diagnosis and surveillance, the term “liquid biopsy” has gained traction but is currently limited by technological challenges in identifying and isolating circulating tumor cells (CTCs), proteins, cell-free DNA (cfDNA), or other nucleic acids. Tumor tissue biopsy, especially in genitourinary (GU) system is sometimes inadequate and requires invasive surgical options, especially for upper tract urothelial cancer. Urine can prove to be “liquid gold” since it may be a more abundant source of tumor-derived material without the background noise; however, urine DNA (uDNA) may be associated with low mutant allele fraction (MAF). Molecular detection of mutations in uDNA requires a sensitive and accurate method of analysis that allows a high depth of sequencing while minimizing artifacts. Several sequencing approaches to address this hurdle using enhanced library preparation techniques such as Tagged amplicon deep sequencing (TAm-Seq), Safe-SeqS, FAST-SeqS, and CAPP-Seq approaches have been developed. Urine biopsy utilizing next-generation sequencing (NGS) can prove useful at all stages of urologic malignancy care, where urine can be collected to aid in clinical decision making through the identification of commonly known mutations, and potentially reduce or avoid all forms of invasive procedures.

## Introduction to Liquid Biopsy

Liquid biopsy refers to any non-tissue specimen, especially body fluids, that can be used to evaluate for tumors in the body, using any of several analytes such as circulating tumor cells (CTCs), proteins, cell-free DNA (cfDNA), or other nucleic acids present in the fluid. Liquid biopsy sources include blood, urine, other body fluids such as stool, saliva, pleural fluid, peritoneal fluid or washings, and cerebral spinal fluid (1), which can minimize the need for expensive, invasive, and sometimes painful tumor tissue biopsies to enable dynamic tumor monitoring. Cell-free tumor DNA or RNA extracted from liquid biopsies can potentially be used in a multiplicity of assays such as next-generation sequencing (NGS) or allele-specific PCR, etc. for the detection of mutations, translocations or copy number alterations, and the expression of specific markers of cancer at the mRNA/small RNA level. These alterations may be used as unique genetic signatures or single-gene tests (1, 2). Detection of somatic alterations and gene expression changes found in bladder tumors through the use of liquid biopsy of urine will be the focus of this review.

Blood is the most commonly described fluid used in liquid biopsy for many types of cancers (3, 4). Blood is the source of CTCs or circulating tumor DNA (ctDNA), circulating tumor RNA (ctRNA), and exosomes, released by tumor tissues, which can be potentially used to detect mutations present in the tumors. The major drawback of using blood as the source of ctDNA is that ctDNA comprises a tiny fraction of cell-free DNA present in the blood, which poses a significant obstacle for accurate and deep sequencing required to detect rare mutations. Moreover, cfDNA is always of low quality and fragmented to the approximate size of a nucleosome (140 bp), and ctDNA is variably present in the blood at earlier stages of cancer (5). So, alternate liquid biopsy approaches such as urine biopsy may be a richer source of tumor-derived material, especially for kidney, prostate, and upper and lower tract urothelial carcinoma, as urine bathes these genitourinary organs. Urine has other unique benefits such as ease of acquisition (does not require trained medical staff), lack of patient discomfort (increased patient compliance), and practically unlimited sample volume, and may have fewer contaminating proteins compared to blood.

Conventional diagnostic and biopsy modalities for bladder cancer include cystoscopy, ureteroscopy with or without biopsy, computed tomography (CT) scans with contrast, which are invasive, inadequate, and not without side effects (6, 7), but given the omnipresence of urine, there are surprisingly few effective liquid biopsy approaches that are widely used. Tavora et al. found that definitive diagnosis cannot be made because of the inadequate tissue in 25% of the renal pelvis or ureteral biopsies. Similarly, Gillan et al. reported significant under detection of carcinoma *in situ* (CIS) and discordance rate between the histopathology of biopsy and resected radical nephroureterectomy (RNU) specimens (7).

## Different Compartments Used in Urine Biopsy

Urine can be used whole (i.e., “neat”) or divided into two compartments useful for biomarker detection: supernatant and pellet. Supernatant consists of partially fragmented cell-free tumor nucleic acids and other tumor-derived materials, while the pellet primarily consists of exfoliated normal and cancer cells, as well as immune cells, debris, and possible bacteria. Several studies have shown that urine supernatant is superior to urine pellet for detection of genetic aberrations in urothelial cancer patients (8, 9). The cfDNA present in the urine supernatant may have higher mutant allele fraction (MAF), due to higher tumor turnover (necrosis/apoptosis) than DNA originating from exfoliated cells due to decreased contamination by normal urothelium and immune cells since those cells are not typically necrotic or apoptotic. Nevertheless, urine pellet has also been successfully used to detect mutations in the upper and lower tract urothelial carcinomas that matched with the mutation profile obtained from tumor tissues of respective patients (10, 11).

## Technical Considerations in Urinary DNA Sequencing

In order to detect very low MAF in urine DNA (uDNA), a sensitive and accurate method of analysis should be used that allows a high depth of sequencing while minimizing artifacts. NGS has the ability to detect rare mutations within a DNA sample but is relatively error-prone due to DNA polymerase errors and read errors during sequencing (12, 13). Although computational methods may identify and filter these variants, these methods are imperfect and may over-filter some true mutations. Use of barcodes or unique molecular identifiers before amplification can separate these errors form real mutation in uDNA (12, 14). It is currently unknown how low the MAF in urine will be, but one might reasonably expect it to potentially be very low after Transurethral Resection of a Bladder Tumor (TURBT), intravesical therapies, or systemic chemotherapy. For instance, prior work shows that there is a mean of 31 mutant copies with a mean of 2018 total copies per mL of urine in patients with bladder cancer recurrence (2). This translates to an *average* MAF of 0.015; many mutations will be present at lower MAF. Although this is low and presents a significant challenge, the problem is even worse in the plasma ctDNA environment.

Several sequencing approaches address this obstacle using enhanced library preparation techniques. Tagged amplicon deep sequencing (TAm-Seq)-based NGS utilizes efficient library preparation and statistical analysis to detect mutations across a gene panel with a detection limit of 0.02% and specificity of 99.99% (15, 16). The Safe-SeqS approach tags each template DNA with unique molecular identifiers prior to amplification to create a unique family of sister molecules descended from the same original molecule resulting in reliable detection of 0.1% MAF with a specificity of 98.9% (12, 17). FAST-SeqS can detect mutation using degenerate bases at 5′ end of the primer that is used as a molecular barcode to label each DNA template (18). CAPP-Seq is an approach that sequences recurrently mutated exons that can detect mutation with allele frequency down to 0.02% with 93% specificity (19). This technique was further improved with unique duplex molecular identifiers and additional informatics filtering to detect mutation allele frequencies as low as 0.004% and specificity of 99.99% (20).

Methods besides NGS are available for liquid or urine biopsy. Droplet digital PCR (ddPCR) and mass spectrometry methods can also be used to detect somatic variants. Droplet digital PCR is based on a water-in-oil emulsion where the tumor or normal DNA is distributed into millions of droplets followed by amplification using TaqMan fluorescence probes which are specific to either the mutant or normal sequences (21). Because the DNA is in limited concentration at the time of droplet formation, droplets tend to either have only one mutant or only one WT allele (or no allele), such that when the template is amplified within the droplet, there is an unambiguous mutant or WT readout within that droplet. This greatly enhances the sensitivity of the method when droplets are sorted by color. The sensitivity of 93% with 100% specificity, with an allele frequency detection limit of 1 in 100,000 molecules have been reported (21).

## Urine Biopsy in Urologic Malignancy Surveillance

Urine has direct contact with bladder tumors, enabling the possibility of relatively large tumor marker quantities (22). Urine can be collected at several diagnostic stages to aid in clinical decision making: prior to presentation as a screening tool; at the time of workup of microhematuria, gross hematuria, or urinary symptoms suggestive of urothelial carcinoma; as a marker of residual disease after treatment; or as a marker of recurrence of urothelial carcinoma (2, 23).

Somatic hotspot mutations within the promoter region of *TERT* are one of the most frequently occurring mutations in different cancers including bladder cancer, of which the most common variants are C>T transition at either of two positions: chr5:12952228 and chr5:1295250, 146 and 124 base-pairs upstream, respectively, of start codon (10, 24, 25). The high frequency of *TERT* promoter mutation has been shown to be prevalent in both muscle-invasive and non-muscle invasive bladder cancer and can be easily detected in urine (10, 25). Kinde et al. analyzed uDNA from 76 patients with non-invasive urothelial carcinoma and showed that mutation in the *TERT* promoter region could be used as a biomarker for early detection of disease in patients being worked up for bladder cancer (11). In addition, they showed that analysis of urinary DNA *TERT* promoter hotspots after TURBT could be used as a marker for recurrent urothelial carcinoma. In another study, *TERT* promoter mutation was significantly associated with 6-month recurrence of pT1 bladder cancer presence of *TERT* mutation increased the risk of recurrence 5-fold, and *TERT* promoter hotspots could be used to non-invasively follow up non-muscle invasive bladder cancer patients after surgery (26). However, these studies were conducted in a small number of patients at a single center and superiority over urine cytology and surveillance cystoscopy still needs to be established for widespread utilization (11, 26).

Similarly, *FGFR3* is mutated in two-thirds of non-muscle invasive bladder cancers [at one of 5 hotspots, with S249C being by far the most common (27)], and the detection of *FGFR3* mutation in urine biopsy was associated with 4-fold higher risk of recurrence (28, 29). In another study, mutation of *FGFR3, RAS*, and/or *PIK3CA* hotspots were analyzed using urine biopsy. At least one of these mutations was present in about 90% of the recurrences, making it feasible to predict the onset of recurrence prior to clinical manifestations (30). Reliance on *FGFR3* mutations is ideal for low grade disease, as these variants are common for these cancers (31). However, the argument can be made that these are the least clinically impactful tumors. Although they recur frequently, almost never progress. Improved biomarkers for low grade/low stage disease are probably not necessary.

Patel et al. showed that the presence of mutations detected by either targeted hotspot panel or copy number alteration detected with shallow whole genome sequencing in uDNA during second neoadjuvant chemotherapy (NAC) cycle was associated with recurrence of bladder cancer with 83% sensitivity and 100% specificity, while the persons without mutation had low recurrence rate with 100% positive predictive value and 85.7% negative predictive value (22). They also revealed that uDNA could be analyzed to assess the tumor evolution during NAC of urothelial carcinoma. This is a highly provocative study but may be impractical to incorporate clinically given that whole genome sequencing was required to detect genetic aberrations in the tumor for 1/3 of the patients.

Several urine biomarkers of urothelial malignancies are FDA-approved for detection and surveillance, five of which use protein-based assays, while UroVysion™ is the only that uses genetic markers (32). UroVysion™ uses exfoliated urothelial cells from urine and analyzes chromosome aneuploidy along with loss of locus 9p21 for the detection of recurrent bladder cancer (33, 34). Meta-analysis showed a sensitivity of 72% and specificity of 83%, with better performance in high-grade urothelial carcinoma, but ~40% sensitivity in low-grade urothelial carcinoma (35, 36). Positive Urovysion™ test in BCG treated patients with superficial bladder cancer was related to treatment failure and high risk of progression to muscle-invasive bladder cancer (37, 38). In comparison, it seems likely that NGS-based methods to detect genetic alterations will be much more sensitive.

The role of non-coding RNAs in bladder cancer has recently emerged in the diagnosis and prognosis of bladder cancer. Two types of non-coding RNAs have been described- small non-coding RNA and long non-coding RNA (lncRNA). The mature forms of these non-coding RNAs act as regulators of gene expression and are never translated into proteins. Micro-RNAs (miRNAs) are an example of a small non-coding RNA subclass that has been investigated extensively. Several studies have reported downregulated or upregulated miRNAs in bladder cancer (39–41). lncRNAs have also been associated with bladder cancer development and progression, although their overall expression and functional significance is still uncertain (42, 43). An essential difference between lncRNAs and miRNAs is their size, with lncRNAs having more than 200 nucleotides. Yazarlou et al. detected the expression levels of four lncRNAs (LINC00355, UCA1–203, UCA1–201, and MALAT1) in urinary exosomes and found that three of them were highly expressed in patients with bladder cancer (44). The combined diagnostic model of lncRNA showed a higher sensitivity (92%) and a higher specificity (91.7%) compared with traditional biomarkers. Seitz et al. identified novel lncRNAs in bladder cancer that act as oncogenic drivers contributing to an aggressive cancerous phenotype through interaction with proteins involved in the initiation of translation and/or post-transcriptional modification of RNA (42, 45).

## Urine Molecular Biomarkers for Precision Medicine

**Figure 1:**
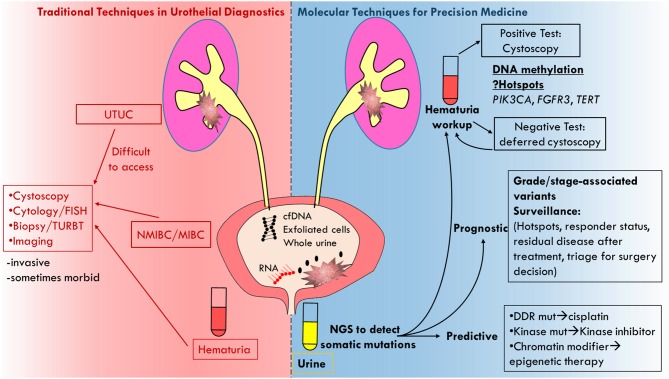
Cartoon depicting molecular analysis of urine biopsy as superior technique to traditional techniques in urothelial diagnostics.

It is important to distinguish how and where urine biopsy could potentially be applied clinically. Prognostic biomarkers such as those described for surveillance are biomarkers that associate with long-term outcome/prognosis, i.e., residual disease status or clinical stage. Predictive biomarkers are associated with or deterministic of response to a particular therapy. Urine biopsy may potentially be used in both settings as we describe below (Figure 1).

### Prognostic Urine Biopsy Applications

Urine biopsy has the potential to be used in monitoring disease response and/or resistance to any therapy that is used for the treatment of bladder cancer including TURBT, intravesical BCG/chemotherapy, systemic chemotherapy or immunotherapy, and radiation. The highest utility of prognostic urine biopsy is probably in the curative/localized setting (46, 47). In particular, urine biopsy could be used to aid in clinical decision-making surrounding cystoscopy in the workup of hematuria (i.e., the initial diagnosis of bladder cancer), follow-up cystoscopy for bladder cancer surveillance, TURBT/bladder biopsy and potentially even radical cystectomy, avoiding unnecessary cost and complications.

#### Initial Workup of Hematuria

American Urological Association (AUA) guidelines recommend performing cystoscopy in patients presenting with gross or microscopic hematuria [with only 3–28% of hematuria patients being diagnosed with bladder cancer depending upon other risk factors such as smoking history, prolonged exposure to chemicals and/or radiation (48)]. Even in patients with risk factors, many negative cystoscopies will be performed to identify a single case, resulting in high diagnostic cost and significant patient burden (49, 50). The safe avoidance of invasive testing/cystoscopy is a desirable outcome for patients and might be achieved with urine biopsy, where test-negative patients could either avoid cystoscopy or undergo deferred cystoscopy. Van Kessel et al. measured DNA methylation in urine biopsy samples and used multivariable analysis of clinical risk factors in hematuria patients to achieve 93% sensitivity and 86% specificity for bladder cancer, thus potentially reducing the need for diagnostic cystoscopy by 77% (50). A limitation of this study was that information on microscopic vs. macroscopic hematuria and cytology were not available and clearly modify the risk of a positive test and of the diagnosis of bladder cancer.

Cxbladder® is urine-based assay that uses reverse transcription quantitative PCR (RT-qPCR) to amplify and detect mRNA level of *CDK1, HOXA13, MDK, IGFBP5*, and *CXCR2* for the detection and surveillance of bladder cancer (51). Cxbladder® *Detect* uses these genotypic factors to detect bladder cancer in hematuria patients with a sensitivity of 82% and specificity of 85% (52). Cxbladder® *Triage*, on the other hand, uses these genotypic factors along with phenotypic factors such as age, gender, frequency of macrohematuria and smoking history to rule out bladder cancer in hematuria patients and have achieved sensitivity as high as 95% and negative predictive value of 97% (53, 54).

UroSEEK®, a massively parallel sequencing-based assay developed by Springer et al., which detects mutations in *FGFR3, TP53, CDKN2A, ERBB2, HRAS, KRAS, PIK3CA, MET, VHL*, and *MLL*, promoter region of *TERT*, and detection of aneuploidy. It has been shown to be effective for the detection of urothelial carcinoma, including bladder and upper tract urothelial cancer (55). UroSEEK was able to detect 83% of bladder cancer cases, which increased to 95% when coupled with cytology, while the sensitivity among upper urothelial carcinoma patients was 75%. Another recent study from Stanford University showed that a high-throughput sequencing-based hybrid capture method for urine tumor DNA detection, uCAPP-Seq, could detect bladder cancer with 84% sensitivity and 96–100% specificity (56).

If these tests were applied to a clinical setting, patients being worked up for the diagnosis of bladder cancer who have positive urine prognostic DNA methylation or mRNA detection tests would be further subjected to cystoscopy, while test-negative patients might be placed into a cystoscopy deferral program. UroSEEK and Cxbladder offer increased sensitivity especially when combined with urine cytology, however; in patients presenting with microscopic hematuria, UroSEEK and Cxbladder missed 30/177 = 16.9% and 7/45 = 15.6% patients with bladder cancer, respectively (Tables 1, 2) (55, 57). Although cystoscopy is an uncomfortable test for patients to undergo, missing a clinically significant bladder cancer which would have been detected cystoscopically is a high diagnostic bar to overcome.

**Table 1:** Initial evaluation for patients presenting with microscopic hematuria or dysuria using the uroSEEK test (55).

	**Bladder cancer *n* = 177**	**No bladder cancer *n* = 393**
UroSEEK positive	147	**28**
UroSEEK negative	**30**	365

*The bold values indicate the number of patients diagnosed incorrectly (false negative or false positive) using respective methods*.

**Table 2:** Initial evaluation for patients presenting with hematuria using the Cx bladder triage and detect test (57).

	**Bladder cancer *n* = 45**	**No bladder cancer *n* = 391**
Cx bladder triage and detect positive	38	**78**
Cx bladder triage and detect negative	**7**	313

*The bold values indicate the number of patients diagnosed incorrectly (false negative or false positive) using respective methods*.

#### Bladder Cancer Surveillance

Recurrences occur in up to 50% of non-muscle invasive bladder cancer patients depending on the stage, multifocality, size, and grade of the tumor, and this necessitates lifelong surveillance cystoscopy in high-risk cases (58, 59), which makes bladder cancer the most expensive cancer that is treated in America (30, 56). Use of urine biopsy in follow up can potentially improve quality of life by reducing the need for invasive testing. Urine biopsy could foreseeably result in cost reduction too, if such a test had good long-term prognostic power (i.e., a “one and done” test), although this has not been rigorously borne out yet. Dudley et al. used urine CAPP-Seq technique to detect mutations in uDNA for the surveillance of bladder cancer after intravesical treatment, being able to detect recurrent cases in overall 91% of patients that included all patients with positive cytology and more than 80% of the patients that cytology missed (56). Kinde et al. analyzed DNA from urine cell pellets using Safe-SeqS technique in the aforementioned study to show that the presence of *TERT* promoter mutation in uDNA can be directly correlated with recurrence (11). As previously mentioned, the detection of *FGFR3, RAS*, and/or *PIK3CA* mutations can also predict recurrence with excellent accuracy (22, 27–30).

Cxbladder®*Monitor* is commercially available that can be used to test recurrent urothelial carcinoma by detecting mRNA level of five urine mRNA biomarkers *IGF, HOXA, MDK, CDC*, and *IL8R* gene expression along with few clinical variables. The overall sensitivity of 91% and a negative predictive value of 96% within 95% CI was observed for this assay and had reduced sensitivity of 86% for low-grade Ta (54, 60).

Cost-effectiveness of such strategies would only be achieved if the cost of testing all patients to avoid cystoscopy in most patients would cost less than performing cystoscopy in all patients in the absence of a test. Other factors would also need to be considered, such as the cost of missing a diagnosis, the cost of working-up a patient with a false-positive result, and potential complications avoided from invasive procedures. These additional costs will vary depending on whether the missed tumor is a high-risk or low-risk superficial bladder cancer or muscle-invasive. Besides, the benefit of a urine biopsy in this clinical scenario would ostensibly be earlier detection of a recurrent tumor, leading to earlier treatment. Early detection of a low-grade recurrence is not likely to bend the clinical destiny of bladder cancer patients, but early detection of a high-risk recurrence might be more meaningful if it resulted in treatment prior to progression to a muscle-invasive state. Therefore, urine biopsy in surveillance might optimally be applied to patients with higher-risk urothelial cancers. It is important to note that a bladder cancer screening test for asymptomatic patients, or even in high-risk populations such as smokers, would be very difficult to effectively achieve given the low incidence of bladder cancer on a population-based scale.

#### Enhanced Diagnosis of Abnormal Bladder Lesions

BCG is well-known to induce inflammatory changes in the urothelium, and often these can be mistaken for CIS or other malignant manifestations, prompting biopsy of suspicious lesions which merely harbor benign inflammatory changes. Urine biopsy could potentially provide an extra diagnostic dimension to triage these abnormal lesions into groups of those meriting biopsy or treatment under anesthesia vs. those which can be observed.

#### Decisions Regarding Radical Cystectomy

NAC is associated with a 30–40% ypT0 rate at the time of radical cystectomy (61–63). There is a significant desire among patients and urologists to avoid radical cystectomy in patients who achieve ypT0 after NAC due to the morbidity, cost, and complications associated with this disease. Clinical assessment of residual disease status after NAC is challenging with high local recurrence rates in patients achieving cT0 states. Meyer et al. in their study reported 28% relapse rate for muscle-invasive disease and 24% relapse rate for non-muscle invasive disease after achieving cT0 status following NAC (64). Similar results have been reported by several multi-institutional studies (65, 66). Therefore, clinical T0 assessment is not equivalent to a pathologic assessment of a ypT0 state (i.e., in a surgical specimen). Urine biomarkers might enhance the accuracy of the staging of residual disease after NAC by detecting small amounts of tumor genetic material for enhanced staging in order to better identify complete responders for cystectomy avoidance algorithms.

### Predictive Urine Biopsy Applications

In addition, urine biopsy might be used as a predictive biomarker similarly to what has been described using ctDNA for lung cancer or other cancers. For instance, ctDNA can be used to identify *EGFR* mutations for treatment assignment to EGFR inhibitors, and similarly can be used to identify the emergence of resistance to these drugs (67). As kinase inhibitors gain traction in the treatment of urothelial carcinoma (68), these agents will likely be applied in earlier settings, and urine biopsy might be used to guide treatment decisions or detect the onset of resistance mechanisms. Afatinib, an irreversible inhibitor of the EGFR family of kinases, was shown to be effective only in platinum-refractory metastatic urothelial carcinoma patients with *ERBB2* and *ERBB3* gene alterations (69). One might envision the use of afatinib in patients with localized cancers whose tumors contain mutations in *ERBB2* or *ERBB3*, which are common in muscle-invasive bladder cancer (70). This could be given in a biomarker-selected and neoadjuvant fashion, whereby patients are selected based on urine biopsy or tissue-based genetic tests. Given the preponderance of FGFR3 alterations in bladder cancer, FGFR3 inhibitors in biomarker-selected patients using a urine biopsy might be a highly desirable path forward.

Additionally, alterations in DNA repair genes are associated with the increased response of bladder cancer patients to NAC and chemoradiation (71–73). These could foreseeably be detected by urine biopsy and used to triage patients into NAC as well.

## Challenges and Future Perspectives

Urine is “liquid gold” for prognosis, diagnosis, and monitoring of tumor evolution after NAC, BCG treatment, or radiotherapy, especially in patients with upper tract urothelial carcinoma where anatomical considerations make accurate staging challenging. Work in this area will continue to evolve and improve until clinical testing is a reality. Urinary biomarkers are low-hanging fruit in the genomics age and given the absence of widely used biomarkers in urothelial cancers, they would fill a significant need for patient evaluations.

Significant challenges need to be considered though. As mentioned, the low MAF is only the first. Tumor DNA present in urine is prone to degradation in the absence of proper storage and transportation from clinic to molecular biology laboratory. Urine biopsy will require new technologies to preserve the integrity and fidelity of these samples. Fixation of tissue introduces well-known artifacts in NGS analyses (74), and this would ideally be avoided in urine biopsy diagnostic media. False positives or negatives will affect the diagnosis, prognosis, and surveillance of urothelial cancer if urine biopsy from the patients is not stored or transported correctly. Although novel devices for collection, storage, and shipment of urine cell pellets have been described (75), little work has been done to identify novel hi-fidelity fixation methods. Immediate processing and frozen storage would likely preserve the integrity and fidelity of DNA in the sample, but this processing method would be challenging for most centers which may not have immediate access to such equipment such as a centrifuge or −80°C freezer. Although advances in sequencing technology and informatics have made sequencing for detection of tumor DNA more feasible and practical, it is still expensive (i.e., not cost-effective) for serial monitoring of tumor evolution after therapy. Moreover, if it were cost-effective, it may not always be clear what a clinician would do with a positive surveillance test with the absence of clinical manifestations—a change in therapy might be needed, but a change to what? It is not clear yet if it would be safe to avoid cystoscopy or cystectomy, for instance. These questions would need to be answered in prospective trials in order to make meaningful and safe changes to the care of biomarker-selected patients.

Should a consensus panel of genes to be sequenced for urothelial carcinoma be used? It may depend on the clinical question. One might envision a predictive test to focus on currently druggable targets, whereas a prognostic test might be a better test if it included non-druggable targets in order to increase the sensitivity of the test. It will be necessary to develop tests that address specific clinical questions in an accurate, precise, and unambiguous manner. Urine biopsy will likely continue to evolve toward higher specificity and sensitivity along with (hopefully) the reduction of associated costs, adding compliance and comfort to patients suspected of having bladder cancer and establishing itself as an integral part of urology or urologic oncology clinics. However, again, it is critical to maintain the development of urine biopsy tests that address a specific and genuine need in the management of urothelial cancer. The market is littered with high performing tests that never gained traction because they did not address a specific clinical need (or at least do not address it unambiguously), were too expensive, or only add an incremental amount of information to the clinical decision-making process. We believe that urine biopsy utilizing NGS-based methods has the potential to significantly enhance clinical decision making for urothelial cancer patients and their care providers in urology, oncology, and pathology.

## Author Contributions

US and AS wrote the manuscript. PA revised and edited the manuscript.

### Conflict of Interest

PA is a part of Advisory board in Janssen and Astra Zeneca. The remaining authors declare that the research was conducted in the absence of any commercial or financial relationships that could be construed as a potential conflict of interest.
